# What Service Characteristics Are Important to Patients Treated in Musculoskeletal Physiotherapy Services: Designing a Discrete Choice Experiment

**DOI:** 10.1002/msc.70175

**Published:** 2025-07-30

**Authors:** Panos Sarigiovannis, Luis Enrique Loría‐Rebolledo, Nadine E. Foster, Sue Jowett, Benjamin Saunders

**Affiliations:** ^1^ School of Medicine Keele University Keele UK; ^2^ Midlands Partnership University NHS Foundation Trust Newcastle Under Lyme UK; ^3^ Health Economics Research Unit University of Aberdeen Aberdeen UK; ^4^ STARS Education and Research Alliance Surgical Treatment and Rehabilitation Service (STARS) The University of Queensland and Metro North Health Brisbane Australia; ^5^ Health Economics Unit Institute of Applied Health Research University of Birmingham Birmingham UK

**Keywords:** discrete choice experiment, PPIE, qualitative methods, semi‐structured interviews

## 1 Introduction

Musculoskeletal (MSK) conditions, which are the single largest contributor to years lived with disability worldwide (Gill et al. 2023), affect an estimated 20.2 million people across the United Kingdom (UK) and are the second leading cause of sickness absence from work (Versus Arthritis 2023). The majority of MSK conditions can be managed in primary care or outpatient services in hospitals; evaluation and treatment by physiotherapists are frequently part of the clinical pathway (Budtz et al. 2021). Patients are assessed by physiotherapists, and if it is appropriate to have follow‐up treatments, these are provided by either a physiotherapist or a physiotherapy support worker.

Physiotherapy support workers, who may also be known as physiotherapy assistants, rehabilitation assistants, technical instructors or physiotherapy technicians, are non‐registered staff who work alongside physiotherapists to provide delegated interventions and responsibilities (Sarigiovannis et al. 2022). The introduction of physiotherapy support worker roles has been driven by global pressures on healthcare systems, including an ageing population, increasing demands on services, workforce shortages, rising costs, and higher patient expectations (Lizarondo et al. 2010; Munn et al. 2013). These roles have become well‐established in countries such as the United Kingdom, Australia, Canada, and the United States, where support workers play a growing part in delivering delegated care across a range of physiotherapy settings.

It has been indicated that patients treated within inpatient hip fracture rehabilitation services have a significant preference for the healthcare professional delivering the follow‐up rehabilitation sessions to be a registered physiotherapist or occupational therapist instead of a support worker (Charles et al. 2018). However, there is a lack of evidence regarding patients' treatment preferences when they are treated in musculoskeletal outpatient physiotherapy services. This led to the design of a discrete choice experiment (DCE) to elicit patients' preferences when treated by physiotherapists and physiotherapy support workers in NHS MSK physiotherapy services. The DCE is part of a mixed methods study which explores the delegation of clinical tasks to physiotherapy support workers (Sarigiovannis et al. 2023).

DCEs are an attribute‐based survey method for measuring benefits (utility). Within healthcare, DCEs have been applied to address a wide range of issues in the delivery of healthcare including measuring and valuing attributes of a healthcare service and identifying the factors that influence choices and decisions of patients, the public and healthcare professionals (Ryan et al. 2001; Soekhai et al. 2019). DCEs are based on the assumption that a service can be described by its characteristics or attributes, and the extent to which an individual values the service depends on the levels of these characteristics (Street et al. 2008). Participants are typically asked to choose between hypothetical service options, each defined by varying combinations of attribute levels, thereby revealing underlying preference structures (Soekhai et al. 2019). For example, Gilbert et al. (2021) used a DCE to examine patient preferences for virtual consultations in orthopaedic rehabilitation. The study found that both service‐related attributes (e.g., therapist familiarity, timing) and patient context (e.g., education, access to equipment) significantly influenced preferences.

An important part in designing a DCE was defining the choice context and deciding what characteristic participants consider important for the choice of service. The aim was to have a sufficiently rich set of attributes and choice contexts, together with enough variation in the attributes levels necessary to produce meaningful behavioural responses about care (Ryan et al. 2008). This short report describes how the DCE attributes and levels were selected, combining data from semi‐structured interviews which formed part of a focused ethnographic study as well as input from a patient and public involvement and engagement (PPIE) group.

## 2 Methods

Development of the DCE, which included selection of the attributes and levels, as well as preparation and testing of the survey instrument, was guided by the input from a PPIE group and the qualitative findings from a focused ethnographic study. The initial meeting and training session was conducted prior to the ethnographic study. First, an initial meeting and training session was conducted with the PPIE group prior to the focused ethnographic study. The latter explored how the use of delegation of clinical tasks from physiotherapists to physiotherapy support workers was informed by the culture within the clinical setting as well as perceptions of, and attitudes about, delegation among physiotherapists, physiotherapy support workers, physiotherapy managers and patients. Ethical approval was granted by the South West—Frenchay NHS Research Ethics Committee and the UK Health Research Authority (REC reference 21/SW/0158, IRAS project 297095). All participants were approached within the recruiting physiotherapy departments and provided informed written consent prior to the observations and interviews that were conducted during the focused ethnographic study (Sarigiovannis et al. forthcoming).

### 2.1 PPIE Group

Five meetings were held with a group of seven patients to develop the DCE: three males and four females. All patients in the group had experience of treatment by a physiotherapist and/or physiotherapy support worker for MSK. The PPIE group was involved in all parts of the study including the research question, study design as well as data analysis and interpretation. Participants were explicitly asked to identify the most important characteristics for a musculoskeletal outpatient physiotherapy service. As participants were not familiar with the DCE methodology, a one‐hour‐training session was provided by the lead author explaining in plain English the DCE methodology, focusing on the function and importance of the attributes or characteristics. Participants had the opportunity to ask questions and at the end of the training session they completed an evaluation questionnaire which indicated that they all had a basic understanding of the DCE concept.

### 2.2 Semi‐Structured Interviews

A focused ethnography was conducted in two MSK outpatient physiotherapy services. The focused ethnographic study included semi‐structured interviews of 19 patients who were treated by physiotherapists and support workers for MSK (Sarigiovannis et al. forthcoming). Table 1 shows the demographic characteristics of the participating patients as well as the reason and mode of their treatment.

**TABLE 1:** Patient participant demographics.

Identifier	Age	Gender	Treated area	Mode of treatment
PIN‐001	70	Female	Hip	One to one
PIN‐003	54	Female	Lower back	Group
PIN‐005	70	Female	Lower back	One to one
PIN‐006	81	Male	Knee	One to one
PIN‐008	74	Male	Knee	One to one
PIN‐010	77	Female	Shoulder	One to one
PIN‐011	70	Male	Lower back	Group
PIN‐012	77	Female	Shoulder	One to one
PIN‐013	74	Male	Hip	One to one
PIN‐015	69	Female	Shoulder	One to one
PIN‐016	82	Male	Hip	One to one
C‐PIN001	74	Female	Lower back	Group
C‐PIN002	92	Female	Shoulder	Group
C‐PIN003	22	Male	Knee	One to one
C‐PIN004	73	Male	Lower back	Group
C_PIN005	46	Male	Knee	Group
C‐PIN006	33	Female	Foot/ankle	Group
C‐PIN007	57	Female	Knee	One to one
C‐PIN008	48	Male	Knee	Group

Patients who participated in the interviews were asked to consider what service characteristics would be important for them if they had to choose between different musculoskeletal outpatient physiotherapy services. Data collection started in March 2022 and was completed in May 2022. Data from the questions pertinent to patient preferences were analysed using a thematic analysis approach which included systematic data coding, generating initial themes from coded and collated data, and refining themes (Braun and Clarke 2020). The qualitative data analysis software QSR NVivo was used to facilitate the analysis. PS coded all data and produced an initial list of themes (attributes), which were reviewed by BS before the final list was produced.

### 2.3 Attributes and Levels

The data from the semi‐structured interviews were shared and discussed with the PPIE group with the objective of compiling a candidate attribute list. Specifically, the group participants were asked to review the list of important service characteristics (attributes) they agreed upon during their first meeting, in light of the qualitative data, and decide if any changes were needed. The revised list of attributes from the qualitative data and the PPIE feedback were then shared with a group of clinicians who formed the Clinical Advisory Group (CAG) of the wider mixed methods study. The following discussion with the clinicians, levels for all attributes were identified, discussed and agreed. The levels for waiting times, travelling distance and number of follow up treatments were chosen with the aim of reflecting actual scenarios that patients might face in the participating clinics. The PPIE group reviewed the final list of attributes and levels before the DCE design was finalised.

## 3 Results

### 3.1 PPIE

Upon completion of the DCE training session and the first meeting related to DCE, the PPIE group members agreed that the service characteristics would be important if they had the opportunity to choose the clinic to attend their musculoskeletal outpatient physiotherapy appointment were continuity of care that is treated by the same clinician; mode of treatment that is treated in a one to one session or in a group with other patients, waiting time for first follow‐up appointment, frequency of follow‐up treatments, number of follow‐up treatments, clinic proximity from home that is travelling distance, parking availability, parking and/or travel costs (to get to the clinic/treatment site), and finally accessibility—disabled access.

### 3.2 Data From the Semi‐Structured Interviews

Most patients mentioned that seeing the same clinician during the follow‐up treatments was an essential service characteristic:

I’d like to keep the same person, you know each time because that way then I think they get to know you and you get to know them so they don’t have to keep referring back to what’s gone on before.

PIN‐012/Patient (female), service 1

Clinic location and travelling distance were listed by the majority of patients in both participating physiotherapy services as important service characteristics:

Location, yes. I would choose somewhere nearest to where I live because I don’t drive very far.

PIN‐005/Patient (female), service 1

The number and frequency of the follow‐up sessions as well as how the treatment is delivered were also highlighted.

I felt it was very important for me to see someone face to face, one to one and as quickly as possible because I was in a lot of pain, I would have gone anywhere.

C‐PIN007/Patient (female), service 2

Most patients were explicit that waiting times would also be considered.

How long you have to wait for your second appointment [NB first follow‐up appointment] would be important. Obviously, when you have a physical problem, you want it resolved as soon as possible.

PIN‐0013/Patient (male) service 1

Finally, patients in both services referred to the importance of parking availability.

Well in my case parking is important, I prefer to go to a clinic where I know there is enough parking available.

C‐PIN‐002/Patient (female), service 2

### 3.3 List of Attributes and Levels

**FIGURE 1:**
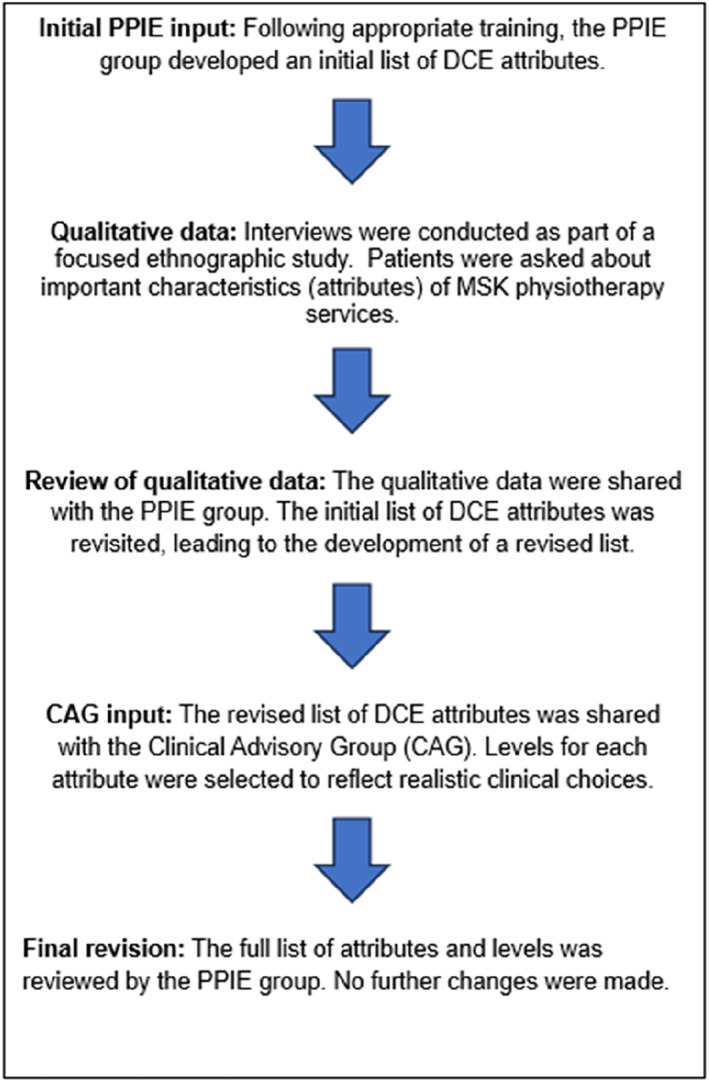
Process for selecting DCE attributes and levels.

After discussing the qualitative data findings, the members of the PPIE group agreed that ‘travel costs’ and ‘accessibility’ would not be included. The aim was to reduce the number of included attributes so that the choice tasks would not become over complicated for participants and reduce the cognitive burden of the resulting choice task. Consequently, seven attributes were included in the DCE. Figure 1 outlines the process undertaken to identify and finalise the DCE attributes and levels. The final list of attributes and levels selected for the DCE are shown in Table 2.

**TABLE 2:** DCE attributes and levels.

Attributes	Description	Levels (coding)
Treating clinician	Who is treating you in your follow‐up sessions? e.g., physiotherapists or physiotherapy assistants/support workers	– Physiotherapists (1) – Physiotherapy assistants/support workers (2)
Waiting times	How long you have to wait to be seen for your first follow‐up session after your initial physiotherapy assessment?	– 2 weeks (1) – 4 weeks (2) – 6 weeks (3) – 8 weeks (4)
Continuity of care	Treating clinician in your follow‐up sessions i.e. if you are seen by the same or different person	Seen by the *same* person (1) Seen by *different* person (2)
Number of follow‐up treatments	If you have follow‐up treatments and if you do, how many?	– 2 follow‐up (1) – 4 follow‐ups (2) – 6 follow‐ups (3) – 8 follow‐ups (4)
Mode of follow‐up treatment	How you have your follow‐up treatments e.g. one‐to‐one or in a group?	– *One‐to‐one* with you and the therapist—*no* exercise equipment (1) – In an *exercise class* (gym) with other patients (2) – *One‐to‐one* with you and the therapist–*with* exercise equipment (*gym*) (3)
Distance to the clinic	How far you have to travel to get to the clinic	– 2 miles (1) – 4 miles (2) – 8 miles (3) – 16 miles (4)
Parking facilities	Availability of parking when attending your physiotherapy appointments	– *Limited* parking (1) – *Ample* parking (2)

## 4 Discussion

One of the known challenges of using a DCE is the need to limit the number of attributes included in the design to avoid overwhelming participants (Green and Srinivasan 1990). Although this study included seven attributes, consistent with the range commonly used in healthcare DCEs (Soekhai et al. 2019), this meant that some attributes identified during the qualitative phase had to be excluded. We aimed to construct a list of attributes such that they captured, directly or indirectly, the most important aspect of a decision. For example, although travel cost was not directly included as an attribute, ‘distance to clinic’ was used as a proxy. However, we do acknowledge that the two are not necessarily equivalent, and this substitution may have influenced how respondents evaluated trade‐offs. These methodological compromises are often necessary in DCE design but can limit the interpretability or generalisability of results, particularly when important dimensions of care are overly simplified or omitted. Coast and Horrocks (2007), Coast et al. (2012), Kløjgaard et al. (2012) and Janssen et al. (2016) highlighted the value of using qualitative methods to help develop DCE attributes for healthcare and the challenges in finding the right balance between the aim in qualitative work to explore and describe, and the reductiveness needed to encapsulate the different aspects of the service within a minimum number of attributes for use in the discrete choice modelling. Kløjgaard et al. (2012) et al. pointed out that the qualitative process can work both as an essential source of valuable information but also as a mean of ensuring the inclusion of stakeholders and key‐persons in an early stage of developing a DCE. Participation of patients and clinicians through the focused ethnographic study as well as the CAG and PPIE groups was instrumental in designing our DCE.

The importance of PPIE inclusion in health and social care research is well reported in the literature as inclusive collaboration between patients, the public and researchers can lead to productive relationships, ensuring that research addresses patient needs (Staniszewska et al. 2017; Blackburn et al. 2018; Aiyegbusi et al. 2023; Arumugam et al. 2023). Shields et al. (2021) highlighted the value of using input from a PPIE group in discrete choice experiments alongside other methods such as a literature review and qualitative research as it may reduce the likelihood of missing attributes by considering a wider perspective. Challenges include the time taken for the process, availability and engagement of the PPIE group members to attend all meetings as well as challenges interpreting attribute terminology and understanding the DCE concept (Shields et al. 2021). We tried to address these challenges by working with our PPIE group in all stages of the study, having regular meetings and providing training when necessary. Blackburn et al. (2018); Arumugam et al. (2023) and Al‐Janabi et al. (2021) emphasised the necessity of prior training for PPIE group members and tailoring the training to the needs of the group to enhance understanding and facilitate engagement and participation. Training was provided to our PPIE group. Consequently, all members of the group developed a basic understanding of the DCE concept and the role of attributes or characteristics, which was essential for their participation, engagement and contributions to the selection of the attributes of our DCE.

The combination of qualitative data and input from a PPIE group and a CAG were essential in the selection of attributes and levels, which would inform the design of the discrete choice experiment. The fact that data from the PPIE group and the focused ethnographic study were almost identical, gives confidence on the robustness of the resulting DCE attributes and levels. Nevertheless, when time and/or resources are scarce, the use of a PPIE group and a CAG alone should be considered when researchers select attributes and levels for a discrete choice experiment within health care as long as the PPIE group participants have experience on the topic under investigation, are fully engaged and have been appropriately trained to have a basic understanding of the DCE and attributes concepts.

## 5 Conclusion

The key service characteristics patients with MSK conditions consider to be important when they are treated by physiotherapists or physiotherapy support workers in NHS MSK outpatient physiotherapy services are continuity of care, mode of treatment, waiting time for first follow‐up appointment, frequency of follow‐up treatments, number of follow‐up treatments, clinic proximity from home and parking availability. These will form the basis of a DCE study in the next steps of this research programme.

## Author Contributions

All authors have made substantial contributions to all of the following: (1) the conception and design of the study, or acquisition of data, or analysis and interpretation of data, (2) drafting the article or revising it critically for important intellectual content, (3) final approval of the submitted version.

## Conflicts of Interest

The authors declare no conflicts of interest.

## Data Availability

The data that support the findings of this study are available on request from the corresponding author. The data are not publicly available due to privacy or ethical restrictions.
